# Histone H4R3 Methylation Catalyzed by SKB1/PRMT5 Is Required for Maintaining Shoot Apical Meristem

**DOI:** 10.1371/journal.pone.0083258

**Published:** 2013-12-12

**Authors:** Minghui Yue, Qiuling Li, Ya Zhang, Yan Zhao, Zhaoliang Zhang, Shilai Bao

**Affiliations:** 1 State Key Laboratory of Molecular Developmental Biology, Institute of Genetics and Developmental Biology, Chinese Academy of Sciences, Beijing, China; 2 University of the Chinese Academy of Sciences, Beijing, China; Institute of Genetics and Developmental Biology, Chinese Academy of Sciences, China

## Abstract

The shoot apical meristem (SAM) is the source of all of the above-ground tissues and organs in post-embryonic development in higher plants. Studies have proven that the expression of genes constituting the WUSCHEL (WUS)-*CLAVATA* (*CLV*) feedback loop is critical for the SAM maintenance. Several histone lysine acetylation and methylation markers have been proven to regulate the transcription level of *WUS*. However, little is known about how histone arginine methylation regulates the expression of *WUS* and other genes. Here, we report that H4R3 symmetric dimethylation (H4R3sme2) mediated by SKB1/PRMT5 represses the expression of *CORYNE* (*CRN*) to maintain normal SAM geometrics. *SKB1* lesion results in small SAM size in *Arabidopsis*, as well as down-regulated expression of *WUS* and *CLV3*. Up-regulation of *WUS* expression enlarges SAM size in *skb1* mutant plants. We find that SKB1 and H4R3sme2 associate with the chromatin of the *CRN* locus to down-regulate its transcription. Mutation of *CRN* rescues the expression of *WUS* and the small SAM size of *skb1*. Thus, *SKB1* and *SKB1*-mediated H4R3sme2 are required for the maintenance of SAM in *Arabidopsis* seedlings.

## Introduction

The shoot apical meristem (SAM) is a self-maintaining structure harboring stem cells which give rise to all of the above-ground tissues and organs in higher plants. The stem cells reside at the center of the SAM, known as the central zone (CZ). The SAM also possesses two other zones, the peripheral zone (PZ) and the rib zone (RZ), which are comprised by the descendant stem cells. The PZ is the source of lateral organs, while the RZ gives rise to the pith of stem [1-3].

Underlying the CZ, there is a region containing about ten cells termed the organizing center (OC), in which a homeodomain transcription factor WUSCHEL (WUS) is expressed to maintain the number of stem cells [4]. In *wus* mutants, SAM is not properly organized in the embryo. In the postembryonic phase, the defective SAM terminates prematurely in flat structures, and new SAMs are initiated and terminated repeatedly [5]. Mature WUS protein migrates to the CZ to activate the transcription of *CLAVATA3* (*CLV3*), which is a marker gene for stem cell identity, by binding to its promoter [6]. 

*CLV3* encodes a 96-amino acid protein with an 18-amino acidic peptide secretory signal in its N-terminal region [7]. After synthesis in the stem cells, CLV3 is processed to a 13-amino-acid arabinosylated glycopeptide, and then secreted into the extracellular space [8]. *clv3* mutants develop enlarged SAMs during both the embryonic and postembryonic phases, and show an enlarged *WUS* expressing domain in the SAM [9,10]. Overexpression of *CLV3* results in a *wus*-like phenotype [11]. Thus, *CLV3* forms a negative feedback loop with *WUS* to maintain the constant number of stem cells. Mutation screening and genetic analysis have identified several receptor-like kinases that play important roles in transmitting CLV3 signal for repression of *WUS* transcription. *CLV1* encodes a transmembrane protein which is comprised of an extracellular leucine-rich repeat domain, a transmembrane domain, and an intracellular Ser/Thr kinase domain [12]. CLV2 has a similar structure but lacks the kinase domain [13]. Both *clv1* and *clv2* mutants have similar phenotypes and *WUS* expression patterns with *clv3* mutants [10]. CLV3 can form a 185 kD complex with CLV1 and bind to the ectodomain of CLV1 directly [14,15].

CORYNE (CRN) is another receptor-like kinase that transmits CLV3 signal. It comprises an N terminal signal peptide, a transmembrane domain, and a Ser/Thr kinase domain. *CRN* defect in *Arabidopsis* results in large SAM size, increased floral organ number, abnormal siliques, etc. [16]. Genetic and biochemical analyses indicate that CRN forms a heterodimer with CLV2 to transmit CLV3 signal, not only because that CRN lacks the extracellular receptor domain and CLV2 lacks the kinase domain, but also because they require each other to localize to the membrane [17-19]. 

CRN also mediates the interaction of CLV1 with CLV2, CRN, and RECEPTOR-LIKE PROTEIN KINASE 2 (RPK2) / TOADSTOOL 2 (TOAD2). RPK2/TOAD2 is an additional receptor kinase that functions in transmitting the CLV3 signal [20,21]. RPK2 doesn’t interact with CLV1 or CLV2 in the absence of CRN, while CLV1 only interacts weakly with CLV2 in the presence of CRN [18,19].

SKB1/PRMT5 is a member of the type II arginine methyltransferase family, which is conserved among many eukaryotic species [22-26]. In human, SKB1/PRMT5 was found to mediate symmetric dimethylation of arginine residues of histone and non-histone protein to regulate chromatin remodeling, gene transcription, pre-mRNA splicing, and protein stability [27-31].. In *Arabidopsis*, we and others have found that SKB1 promotes flowering and responses to salt stress through controlling both gene transcription and pre-mRNA splicing [32-35], and regulates circadian rhythms by regulating alternative splicing [36]. 

In this study, we show that the seedlings of *skb1* mutant plants grew slower and smaller than WT plants. A detailed morphological analysis revealed that the SAM size of *skb1* was significantly reduced. We found that the expression levels of *WUS* and *CLV3* were down-regulated in *skb1*, and up-regulated *WUS* expression could rescue the small SAM size of *skb1*. We also found that SKB1 and histone H4R3sme2 were associated with the chromatin of the membrane kinase gene *CRN*, and did not repress *WUS* expression directly. Introduction of *CRN* mutation into the *skb1* mutant could rescue both *WUS* expression and the small SAM size.

## Results

### SAM defects of *skb1* mutants

**Figure 1:**
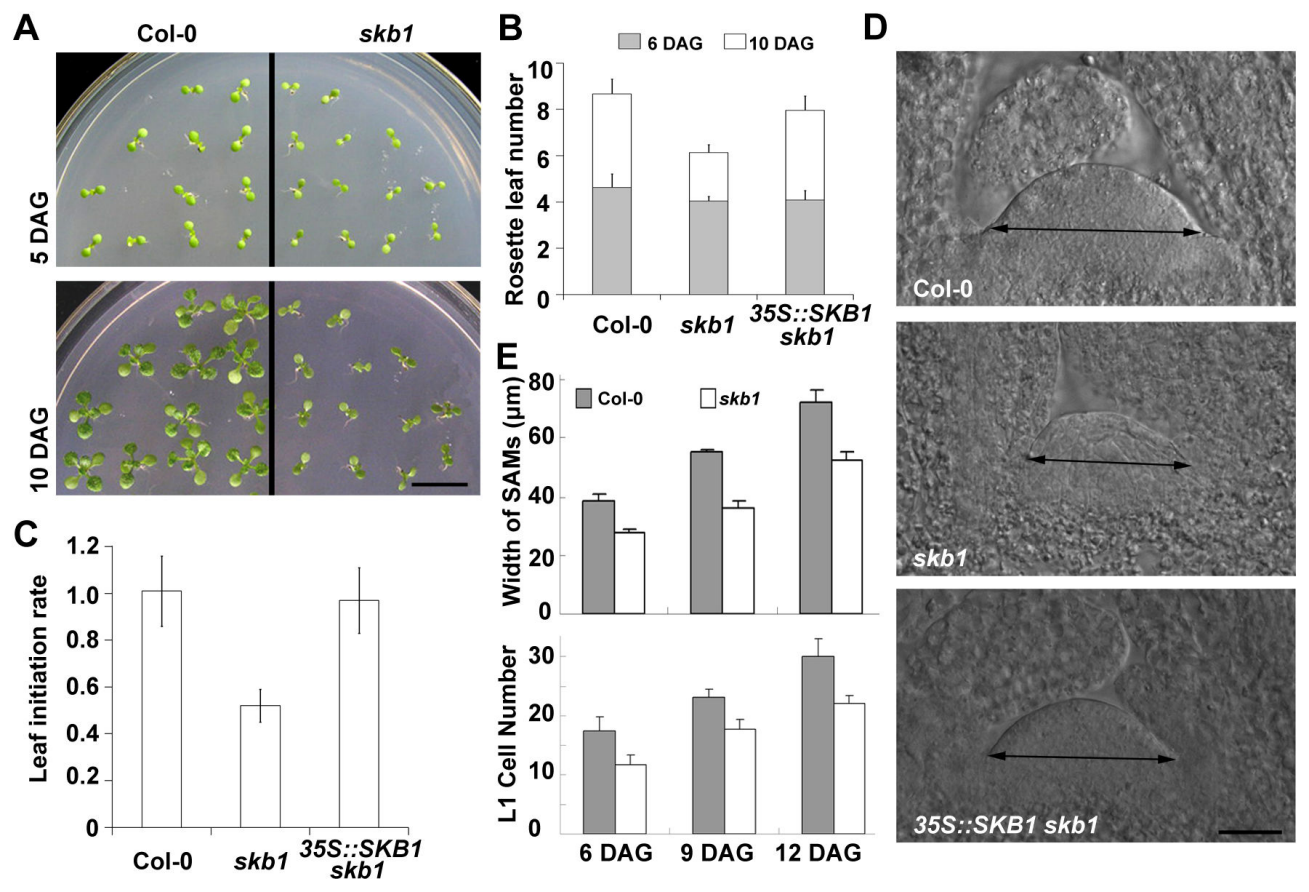
Phenotypes of growth rates and SAM size in *skb1* seedlings. (A) Comparison of seedling size of Col-0 and *skb1* grown on MS medium at 5 DAG and 10 DAG. Scale bar = 1 cm. (B) and (C) Comparison of rosette leaf growth rate of Col-0, *skb1* and 35S*::*SKB1 *skb1* plants grown on MS medium. Leaf initiation rate is calculatedas the number of new leaves produced per day between 6 DAG and 10 DAG. Data represent the means ± SE (n=36). (D) Comparison of SAM size of Col-0, *skb1* and 35S*::*SKB1 *skb1* plants grown on MS medium at 6 DAG. Scale bar = 20 μm. (E) Quantitative analysis of SAM size (upper panel) and Layer 1 cell number (lower panel) of Col-0 and *skb1* plants grown on MS medium at 6 DAG, 9 DAG and 12 DAG. L1 means layer 1 of SAM. More than 13 SAM sections were measured for size and counted for L1 cell number under microscope for each data point. Data represent the means ± SE.

*skb1* mutants display many characteristics during their development, including curled and darker-colored leaves, late flowering, hypersensitivity to salt stress, alteration of circadian clock, etc. [32-36]. We also found that the *skb1* seedling size was smaller when compared with the wild type Col-0 seedlings. At 10 days after germination (DAG), the size of *skb1* seedlings was significantly smaller than that of Col-0. Interestingly, the difference between seedling sizes at 5 DAG was minimal (Figure 1A). We compared mature seeds of *skb1* and Col-0 and found that there were no obvious morphological defects, nor was the mass of the *skb1* seeds decreased (Figure S1). This indicates that the small seedling was a growth defect in *skb1* mutants, rather than a developmental defect of the seeds. Moreover, the *skb1* plants grew to a normal size at later developmental stages (Figure S2). 

To fully characterize the growth defect of *skb1* seedlings, we examined the leaf initiation rates and found that *skb1* leaf initiation was decreased about 50% compared with that of Col-0 (Figure 1B and 1C, Figure S3). This decrease suggests that the *skb1* mutants may have lower SAM activity than Col-0. To verify this notion, we measured the SAM size of the seedlings at different post-embryonic stages to see whether the size was relative to the activity of SAM. We found that the SAM size in *skb1* plants was smaller than in Col-0 at 6 to 12 DAG (Figure 1D and 1E). Under microscope, a typical three layers of SAM outer cells (L1, L2, L3) could be observed clearly in structure without difference between Col-0 and *skb1* mutant. Consistent with the SAM size, L1 cell number in *skb1* plants was also less than in Col-0 at different post-embryonic stage (Figure 1E, lower panel). When we introduced a *35S::SKB1* construct into the *skb1* mutants, both the leaf initiation rate and SAM size phenotypes were reversed (Figure 1B to 1D). These data indicate that *SKB1* plays an important role in maintaining SAM size and in regulating SAM activity in *Arabidopsis* seedlings.

### Genetic interaction between *SKB1* and *CLV* genes

**Figure 2:**
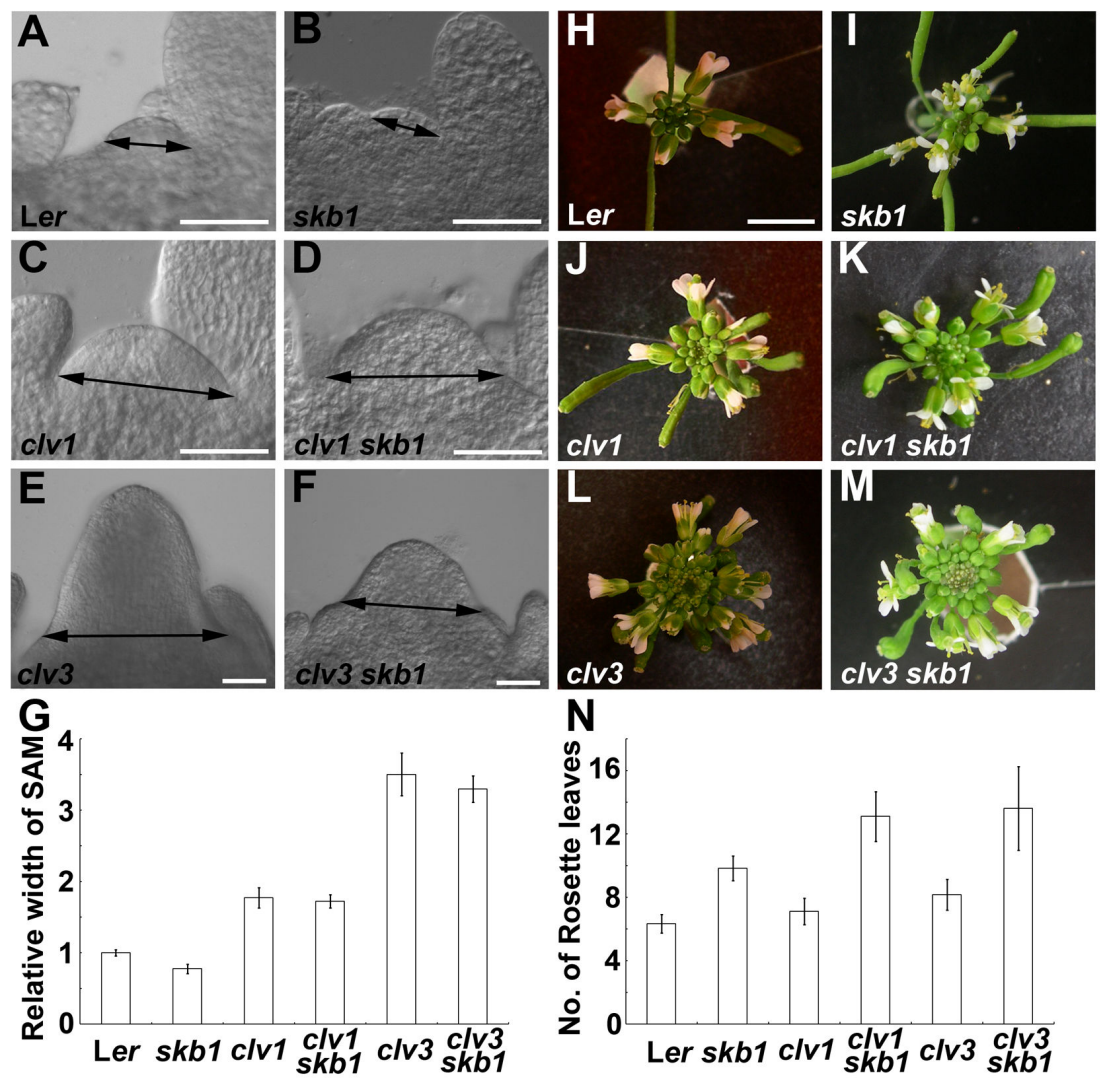
Phenotypes of *clv1 skb1* and *clv3 skb1* double mutants. (A) to (G) Comparison of the SAM size of L*er* (A), *skb1* (B), *clv1* (C), *clv1* *skb1* (D), *clv3* (E), *clv3* *skb1* (F) plants grown on MS medium at 9 DAG. Data in (G) represent the means ± SE (n≥12). Scale bar = 50 μm. (H) to (M) Top view of inflorescences of L*er* (H), *skb1* (I), *clv1* (J), *clv1* *skb1* (K), *clv3* (L), *clv3* *skb1* (M) at 5 weeks (H, J and L) or 7 weeks (I, K and M). Scale bar = 5 mm. (N) Comparison of the flowering time of L*er*, *skb1*, *clv1*, *clv1* *skb1*, *clv3* and *clv3* *skb1*. Bars represent mean values standard error deviation of rosette leaf number at bolting. For each line, 18 plants were scored.

Unlike the small SAM size of *skb1* mutants, *clv* mutants showed enlarged SAMs. To investigate whether a genetic interaction exist between *SKB1* and *CLV* genes, we crossed *skb1* (L*er* background) with *clv1-1* and *clv3-2*. The SAM sizes of *clv1 skb1* and *clv3 skb1* at 9 DAG were much larger than *skb1* single mutants, while they showed only a slight difference from *clv1* and *clv3*, respectively (Figure 2A to 2G). The sizes of the inflorescence meristems of *clv1 skb1* and *clv3 skb1* were also increased markedly compared with *skb1* (Figure 2H to 2M). We also over-expressed *SKB1* in *clv1-1* and *clv3-2* by driving *SKB1* under the control of the cauliflower mosaic virus *35S* promoter. The defects of the inflorescences, flowers, and siliques of *clv*s didn’t show any detectable rescue (Figure S4 and Data not shown). 

In addition to the stem cell defects of *clv1 skb1* and *clv3 skb1*, we noticed that these plants exhibit late flowering. This late flowering phenotype is similar to *skb1* and distinct from *clv1* and *clv3* (Figure 2N). Thus, SKB1 appears to maintain the shoot meristem upstream of *CLAVATA* genes, or regulate the process in a parallel pathway.

### *SKB1* is required for the expression of *WUS* and *CLV3*

**Figure 3:**
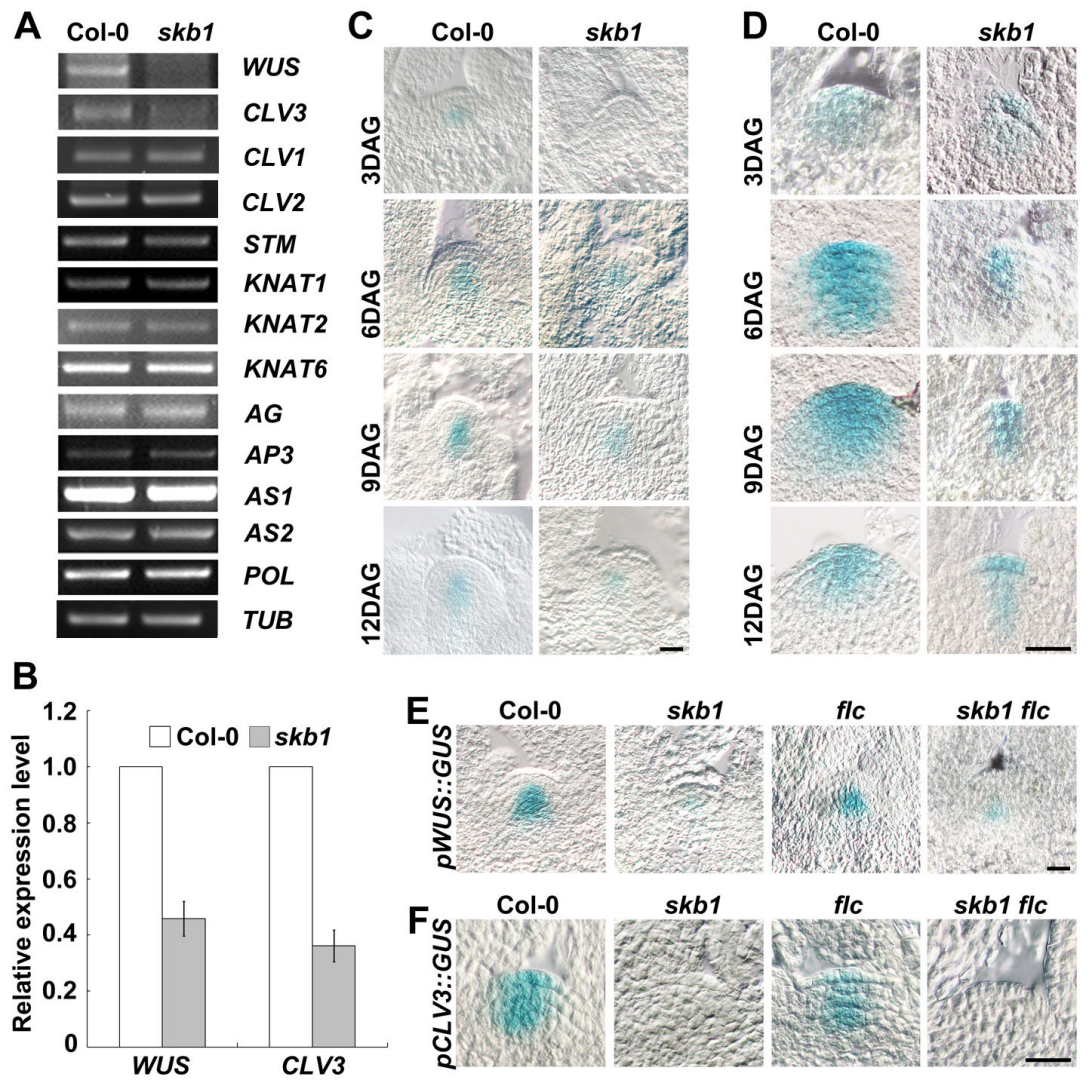
Differences in expression profiles between Col-0 and *skb1* plants. (A) Semi-quantitative RT–PCR analysis of known meristem regulators. TUBULIN (TUB) was the loading control. Three biological replicates were performed with similar results. (B) Quantitative real time PCR analysis of the expression of *WUS* and CLV3 in Col-0 and *skb1* seedlings and normalized with *ACTIN2* expression. Data represent the means ± SE of three independent experiments. (C) and (D) Staining results of GUS activity assays of pWUS*::GUS* (C) and *pCLV3::GUS* (D) transgenic lines in Col-0 and *skb1* backgrounds, respectively. Materials used were seedlings grown to 3, 6, 9 and 12 DAG on MS medium. (E) and (F) Staining results of GUS activity assays of pWUS*::GUS* (E) and *pCLV3::GUS* (F) transgenic lines in Col-0, *skb1*, *flc*, and *flc* *skb1* backgrounds, respectively. Materials used were seedlings grown to 5 DAG on MS medium. Scale bar = 20 μm.

SAM and shoot apical stem cell maintenance is primarily regulated through two pathways. *WUS* and *SHOOT MERISTEMLESS* (*STM*) play the primary roles in each pathway [10,37,38]. To investigate the molecular mechanism underlying the SAM phenotype in *skb1* mutants, we examined the transcript levels of key genes regulating shoot meristem maintenance. Among the genes we tested, *WUS* and *CLV3* were found down-regulated in the *skb1* mutants, but *STM* and other genes were not altered significantly (Figure 3A and 3B). We also used the GUS assay to investigate the localization and dynamic transcription levels of *WUS* and *CLV3*. At 3 DAG, the expression of *WUS* and *CLV3* was quite low in both Col-0 and *skb1*. At 6 DAG and during a few following days, the expression of *WUS* and *CLV3* was lower in *skb1* than in Col-0 (Figure 3C and 3D), but the localizations of *WUS* and *CLV3* were not changed in the *skb1* mutants. These data suggest that *SKB1* is involved in the WUS-CLV feedback loop to maintain the stem cells.

### *SKB1* regulates *WUS* and *CLV3* expression in an *FLC*-independent manner

In *Arabidopsis*, flowering is associated with the transition of SAMs into inflorescence and floral meristems. Flowering time is regulated mainly by four pathways. These are photoperiod pathway, vernalization pathway, gibberellins (GA) pathway, and autonomous pathway [39]. FLOWERING LOCUS C (FLC) is a key regulator of flowering time control, and integrates the autonomous pathway and vernalization pathway. As a member of autonomous pathway genes, *SKB1* works upstream of *FLC* and regulates the transcription of *FLC* directly [33]. We wondered whether the increased expression level of *FLC* in *skb1* mutants was responsible for the small SAM size, so we introduced *pWUS::GUS* and *pCLV3::GUS* to *flc* and *flc skb1* mutants. We found that the expression levels of both *WUS* and *CLV3* in *flc skb1* double mutants were similar to those in *skb1* and much lower than in *flc* or Col-0 plants (Figure 3E and 3F). These results suggest that *SKB1* regulates the expression of *WUS* and *CLV3* in an *FLC*-independent manner.

### Over-expression of *WUS* enlarges the SAM size of *skb1*

**Figure 4:**
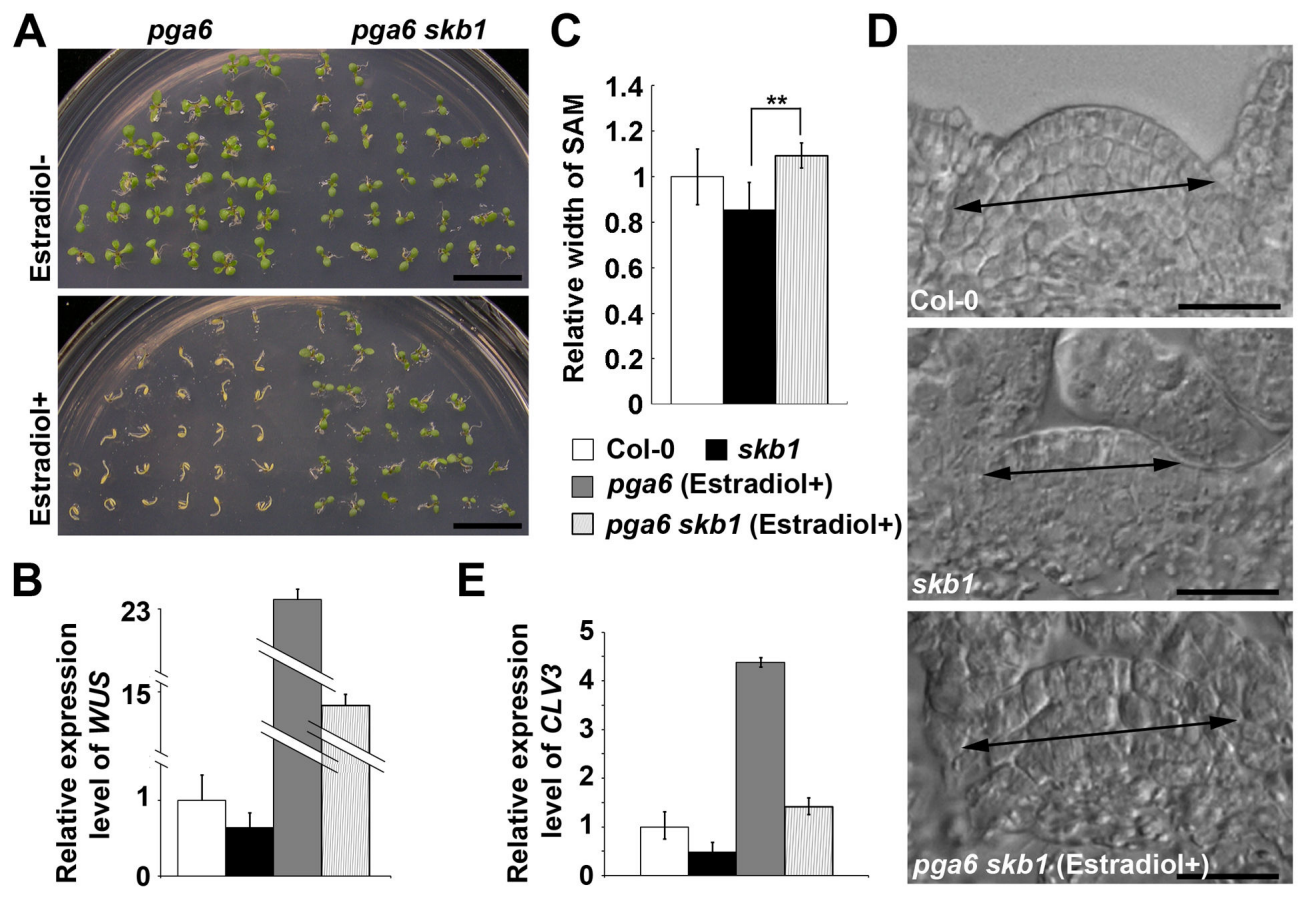
Up-regulation of *WUS* increases the SAM size of *skb1*. (A) Phenotype of *pga6* and *pga6* *skb1* with and without 17-β-estradiol treatment, grown on MS medium at 6 DAG. Scale bar = 1 cm. Estradiol+: treated with 17-β-estradiol; Estradiol-: no treated with 17-β-estradiol. (B) and (E) Quantitative real time PCR analysis of *WUS* (B) and CLV3 (E) expression in Col-0, *skb1*, *pga6* with 17-β-estradiol treatment, and *pga6* *skb1* with 17-β-estradiol treatment. Data represent the means± SE of three independent experiments. (C) and (D) Comparison of the SAM size in Col-0, *skb1*, and *pga6* *skb1* with 17-β-estradiol treatment. Data in (C) represent the means ± SE (n=12), *P* < 0.01. Scale bar in (D) = 20 μm.

 To explore whether a low expression level of *WUS* is the major cause for a small SAM size in *skb1*, we crossed *skb1* with the *pga6-1* mutant to generate a *pga6-1 skb1* double mutant. *pga6-1* is a gain-of-function mutant in which the expression of *WUS* can be induced by application of 17-β-estradiol [40]. In the absence of the inducer compound 17-β-estradiol, the growth of *pga6-1 skb1* seedlings was almost the same as that of *skb1* (Figure 1A and 4A). While in the presence of 17-β-estradiol, the growth of *pga6-1 skb1* seedlings was slightly repressed but not as repressed as in *pga6-1* (Figure 4A). Interestingly, the SAM size was much larger in *pga6-1 skb1* and, even larger than that of Col-0 (Figure 4C and 4D). We examined the expression of *WUS*, and found that it was increased to a much greater extent in *pga6-1* and *pga6-1 skb1* in the presence of 17-β-estradiol than Col-0 and *skb1* (Figure 4B). In the presence of the inducer, the expression level of *CLV3* in *pga6 skb1* double mutant was also higher than in Col-0 (Figure 4E). These results imply that the SAM defect in *skb1* seedlings was correlated with the reduction of the *WUS* expression level.

### SKB1 is required for the deposition of histone H4R3 symmetric dimethylation in *CRN* chromatin

To explore whether *WUS* is a direct target of SKB1, we performed chromatin immunoprecipitation (ChIP) assays using 12-day-old seedlings as materials with an anti-SKB1 antibody. Ten regions covering the promoter and the gene body of *WUS* were tested by PCR. Region c of *WUS* contains the critical *cis* element for the expression boundaries of *WUS* transcription in the shoot apical stem cell niche [41]. The ChIP results revealed that SKB1 could not pull down any regions of the promoter or the gene body of *WUS* (Figure S5). Given that the *WUS* expression cells were less than 1% of the whole seedling, we also collected the inflorescence tip of *clv1* and *clv1 skb1* which have enlarged SAM and much more apical stem cells to perform the experiment, and we got the same results (data not shown). Thus, SKB1 does not regulate the transcription level of *WUS* directly. 

**Figure 5:**
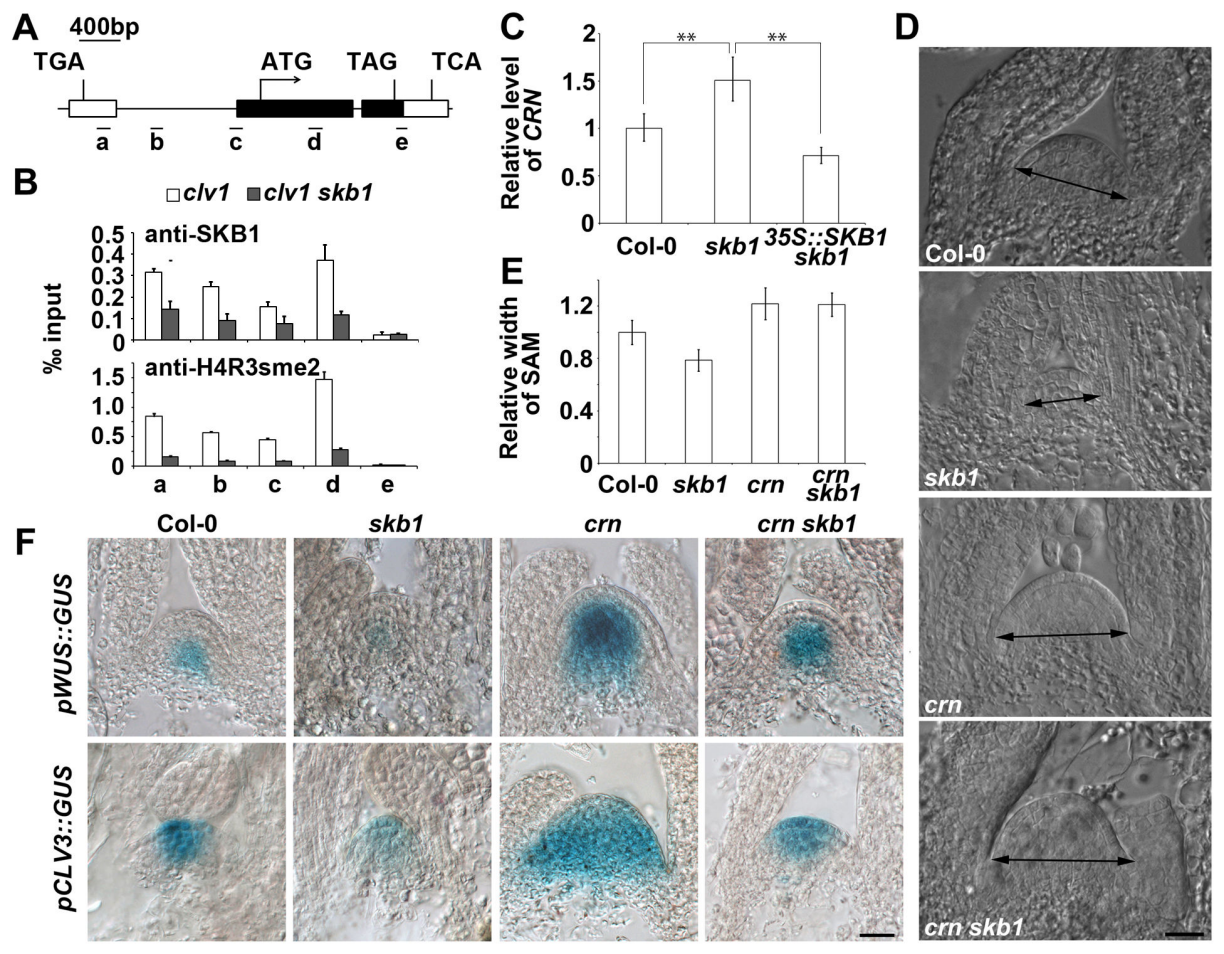
CRN is a major target of SKB1 in regulating SAM size. (A) A diagram of the *CRN* gene structure, with bars representing the a-e regions examined by ChIP. The *CRN* open reading frame was shown by black boxes, while the exons of its neighbor genes (the left is At5G13300 and the right is At5G13280) were shown by white boxes. (B) The ChIP assay for *CRN* was performed with antibodies against SKB1 and H4R3sme2. Chromatin extracted from 12-day-old seedlings of Col-0 grown in soil, and *skb1* mutant chromatin as a control. Data represent triplicate quantitative real time PCR measurements of immunoprecipitated DNA, and the input represents chromatin before immunoprecipitation. Error bars represent relative SD of ChIP data. (C) Quantitative real-time-PCR analysis of the relative expression levels of *CRN* in Col-0, *skb1* and 35S*::*SKB1 *skb1* seedlings and normalized with ACTIN expression. Data represent the means ± SE of three independent experiments, *P* < 0.01. (D) and (E) Comparison of SAM size in Col-0, *skb1*, *crn*, and *crn* *skb1* plants grown on MS medium to 9 DAG. Data in (E) represent the means ± SE (n≥11). Scale bar in (D) = 20 μm. (F) Staining results of GUS activity assays of pWUS*::GUS* and *pCLV3::GUS* transgenic lines in the Col-0, *skb1*, *crn*, and *crn* *skb1* backgrounds, respectively. Materials used were seedlings grown to 9 DAG on MS medium. Scale bar = 20 μm.

Through ChIP-sequencing experiments using anti-SKB1 antibody (data not shown), we found that a receptor-like kinase gene named *CRN* could be a direct target of SKB1. Previous studies have proven that CRN forms a heterodimer with CLV2 to transmit CLV3 signal [18,19]. Mutation of *CRN* resulted in a larger SAM [16]. To confirm the ChIP-seq data, we performed a ChIP (-qPCR) analysis with anti-SKB1 and anti-H4R3sme2 antibodies. SKB1, as well as H4R3sme2 was significantly enriched in the promoter and first exon but not the 3’ end of *CRN* (Figure 5A and 5B). To date, H3R3sme2 was found as a suppressor code for transcription in *Arabidopsis* [33,34]. To confirm that *CRN* was a target gene of SKB1, we checked the expression level of *CRN* in *skb1* mutants, and found that *CRN* was up-regulated in *skb1* (Figure 5C). These results indicate that SKB1 regulates the expression of *CRN* through altering the modification levels of histone mark H4R3sme2 in *CRN* chromatin.

To confirm that the increased expression of *CRN* was the direct cause of the decreased SAM size in *skb1*, we quantified SAM sizes from the *crn skb1* double mutant. At 9 DAG, the SAM size of *crn skb1* was comparable to that of *crn*, providing direct evidence that the small SAM size of *skb1* was rescued by *crn* mutation (Figure 5D and 5E). We also determined *WUS* and *CLV3* expression levels in *crn skb1* and found that both were higher than in the *skb1* single mutant, even than in Col-0, though not as high as in *crn* (Figure 5F). Thus, SKB1 regulates SAM size by methylating H4R3 and controlling the expression of *CRN*.

## Discussion

In this study, we found that the protein arginine methyltransferase SKB1 was functionally involved in the maintenance of SAM in *Arabidopsis*. SKB1 associated with the chromatin of the gene *CRN*, mediated the symmetric dimethylation of histone H4R3, and repressed the transcription of *CRN*. The *WUS* and *CLV3* expression were down-regulated in *skb1* mutants because of the increased *CRN* level, and thus resulted in the smaller SAM size. Our results reveal a previously unknown function of SKB1 in regulating SAM size through the major WUS-CLV signaling pathway.

### SKB1 is important for the maintenance of the SAM

To date, two parallel pathways have been identified to play critical functions in maintaining shoot meristems. The homeodomain transcription factors WUS and STM regulates each pathway, respectively [10,37,38]. It is thought that the WUS-CLV pathway maintains a constant stem cell number, while *STM* pathway prevents differentiation. *WUS* is required for the expression of the stem cell marker gene *CLV3*. CLV3 works as a ligand, binding to the membrane localized receptor-like kinases to restrict the expression of *WUS*. Expression level changes of these genes alter the SAM and seedling phenotypes. We found that the expression of *WUS* was decreased in the *skb1* mutant in which the modification levels of H4R3sme2 reduced, which is not consistent with that histone H4R3sme2 is a suppressor code for transcription if *WUS* is a direct target of SKB1 [33,34]. Further experiments found that SKB1 did not associate with the chromatin of *WUS* (Figure S5), and SKB1-mediated H4R3sme2 directly repressed the transcription level of *CRN*, which is an important member of WUS-CLV pathway. In *skb1* mutants, the expression level of *CRN* was increased, resulting in decreased *WUS* and *CLV3* transcription levels, and small SAM size. It has been reported previously that the *WUS* expression was regulated by several histone modifications, such as H3K27me3, H3K4me3, H3K9me2 and H3K9ac [42,43], our data highlighted the function of histone arginine methylation in controlling the *WUS* expression and SAM size maintenance. 

Although SKB1 can regulate alternative RNA splicing [32,34,36], we did not find any significant pre-mRNA splicing defects of *WUS*, *CLV3* or *CRN* (data not shown). Rather, we noted the transcription level alternation.

Another major pathway that control SAM maintenance is the homeobox genes of the *KNOX* family. One of these, *STM* is reported to distinguish the SAM from lateral organs. *STM* is expressed throughout most of the SAM. It prevents differentiation by restricting the expression of *ASYMMETRIC LEAVES1* (*AS1*) and *ASYMMETRIC LEAVES2* (*AS2*) to the domain where lateral organs will initiate [44,45]. *AS1* and *AS2* are necessary to ensure leaf organs initiate, and have the proper structure and shape [46,47]. In our study, we noticed that the rosette leaf initiation rate of *skb1* was slower than that of WT plants, while the expression of *KNOX* genes (such as *STM*, *KNAT1*, *KNAT2*, and *KNAT6*) as well as *AS1*, *AS2* were not altered. Further study will elucidate how the lower rosette leaf initiation rate occurred in *skb1* mutants.

### Relationship between seedling and SAM size

In this paper and our previously reports, we noticed the mutation of *SKB1* result in smaller organ size and reduced grow rate in *Arabidopsis* (Figure 1 and Figure S2) [33,34]. Similar growth defects were reported in other eukaryotic species. In fission yeast, *Schizosaccharomyces pombe*, cells carrying a *skb1* null mutant were less elongated than WT and exhibit a moderate growth defect [48]. Further studies show that SKB1 is a component of the morphology control branch of the Ras signaling cascade, and negatively regulates mitosis [48,49]. In animals, *SKB1*/*PRMT5* interacts with different transcription factors and chromatin modifying enzymes and mediates methylation of nuclear and cytoplasm proteins to modulate cell growth, transformation, apoptosis, and organelle biogenesis [50]. These results indicate that *SKB1*/*PRMT5* plays a conserved role in regulating growth among different species.

We obtain several lines of evidence that *CRN* is an important target of SKB1 in regulating SAM maintenance in *Arabidopsis*. Mutant of *CRN* could rescue specifically the small size of *skb1* mutants, but not seedling size (Figure S6), which indicates that in addition to the smaller SAM size, there might be other factors that lead to the smaller size of the seedlings. Firstly, though the SAM size of *crn skb1* was almost the same as *crn*, the expression level of *WUS* and *CLV3* was lower than that of *crn*, which may impact seedling growth. Secondly, the root length of *skb1* mutants was reduced compared with Col-0 [34]. The shorter roots caused by *SKB1* lesion might create a deficit in providing sufficient nutrients. Thirdly, *skb1* mutants are hypersensitive to salt stress [34] and the root stem cells are hypersensitive to DNA damage (unpublished data), which may also impact seedlings grow and render the plants much weaker and smaller. Collectively, our studies revealed a *SKB1*–*CRN* regulatory mechanism mediated by H4R3sme2 modification that plays an important role in SAM geometry. 

### SAM maintenance and flowering-time control

The SAM is formed during seed development, and gives rise to all of the above-ground tissues and organs including leaves, stems, flowers, and so on. With transition from vegetative to reproductive growth, the SAM transitions to become the inflorescence and floral meristems. Floral meristem identity factors such as *LEAFY* (*LFY*) and *APETALA1* (*AP1*) are activated by flowering-time control genes such as SUPPRESSOR OF OVEREXPRESSION OF CONSTANS 1 (SOC1) and *FLOWERING LOCUS T* (*FT*) [39]. Previous studies reported that the floral homeotic gene AGAMOUS (AG) repressed *WUS* expression resulted in termination of meristem growth during flower formation [51]. This indicates that flowering is connected with SAM termination. In this study, we noticed that flowering time suppressor *FLC* did not regulate the expression of *WUS* and *CLV3* (Figure 3E and 3F), in spite of the pivotal role of the *FLC* in regulating flowering in *skb1* mutants [33]. In addition, mutation of the *CLV* genes in *skb1* mutant background did not change the flowering time of *skb1* (Figure 2N). This means that flowering-time control is connected with the formation of inflorescence and floral meristems and termination of the SAM, but does not regulate SAM maintenance genes like *WUS* or *CLV*s directly. 

## Materials and Methods

### Plant material and growth conditions

The *Arabidopsis thaliana* ecotypes Col-0 and L*er* were used as wild type plants in this study. *skb1* (Salk_065814), *35S::SKB1 skb1*, *flc* (*flc-3*), *flc skb1*, *clv1-1*, *clv3-2*, *crn* (*sol2-1*), *pga6-1*, *pWUS::GUS* Col-0, and *pCLV3::GUS* Col-0 were described previously [9,33,52,53]. *skb1* (L*er* background) was generated by crossing *skb1* with L*er* and back crossing to L*er* three times. *35S::SKB1* L*er*, *35S::SKB1 clv1-1*, *35S::SKB1 clv3-2* were transformed using construct *35S::SKB1*, as reported previously [33]. 

Plants were grown on MS medium or in soil. Seeds were surface sterilized with 70% ethanol for 1 minute, 15% sodium hypochlorite for 15 minutes, and washed four times with sterile water. Sterilized seeds were sown on MS medium. To promote germination, seeds on MS medium were stratified at 4 °C in darkness for 3 days and then transferred to a culture room at 22 °C under long-day (16 hours light/ 8 hours dark) conditions. 

### RNA extraction and RT-PCR analysis

Total RNA was extracted using Trizol reagent (Invitrogen) from the SAM material grown in soil to 5 DAG (Figure 3B), or grown on MS medium to 6 DAG (Figure 4) or 9 DAG (Figure 3A and 5C). cDNA synthesized using a template of 4 μg RNA using Super Script III (Invitrogen) reverse transcriptase. Semi-quantitative and quantitative real-time PCR were performed, *TUBULIN* and *ACTIN2* were used as endogenous expression control in semi-quantitative and quantitative real-time PCR, respectively. The primers used are listed in Table S1.

### GUS staining

β-glucuronidase (GUS) activity detection was performed following Scarpella et al. [54]. With modification that both potassium ferro- and ferricyanide to 5 mM were added and incubation time of 6 hours was used in all experiments except in Figure 3F, which had a 1 hour incubation time.

### SAM observation and measurement

For SAM size measurement, seedlings with indicated age and genotype were fixed with 4% PFA for 15 minutes, washed by PBS, then were incubated overnight at 4°C in 10% glycerol. Seedlings were embedded in O.C.T. Compound (Tissue-Tek) and were longitudinally sectioned into 20 μm serial slices. These slices were then observed under a differential interference contrast (DIC) microscope. The middle slice for each seedling which had the clearest SAM shape was taken to measure SAM size and L1 cell numbers.

### ChIP and ChIP-sequencing

12-day-old seedlings grown in soil were analyzed in ChIP assays as described previously [55] with antibody anti-SKB1 [33], and inflorescence tips for ChIP-PCR analysis with antibodies anti-SKB1 and anti-Histone H4 (symmetric di methyl R3) antibody (ab5823). Primers used for PCR are listed in Table S1.

 For ChIP-squenece, 2 g of 12-day-old *Arabidopsis* whole seedlings were used. Polyclonal anti-SKB1 antibody was used with BSA-blocked Protein G agarose beads (Millipore) to immunoprecipitate the SKB1-DNA complex. *skb1* mutant seedlings grown under the same condition were used as negative control following the same assay procedure. 

The ChIP-sequencing library was constructed according to Illumina's instructions (www.illumina.com). Sequence reads were mapped to the unmasked *Arabidopsis* genome (TAIR8 build) using the SOAP2.20 software, allowing at most two mismatches at any position. Enrichment was then calculated in each valid base pair by comparing using UCSC Genome Browser. Peak region was valuated using the MACS 1.3.6 software.

### Accession Numbers

Sequence data from this article can be found in the Arabidopsis Genome Initiative or GenBank/EMBL databases under the following accession numbers: *SKB1*, At4G31120; *WUS*, At2G17950; *CLV1*, At1G75820; *CLV3*, At2G27250; *STM*, At1G62360; *KNAT1*, At4G08150; *KNAT2*, At1G70510; *KNAT6*, At1G23380; *AG*, At4G18960; *AP3*, At3G54340; *AS1*, At2G37630; *AS2*, At1G65620; *POL*, At2G46920; *TUB*, At5G62690; *FLC*, At5G10140; *CRN*, At5G13290; *ACTIN2*, At3G18780.

## Supporting Information

Figure S1 **Comparison of *skb1* and Col-0 seeds.** (A) Morphology of *skb1* and Col-0 seeds.
(B) Comparison of weight per thousand seeds of *skb1* and Col-0.
(TIF)

Figure S2 **Phenotypes of *skb1* in different growth stage.** (A) to (D) Sizes of *skb1* compared with Col-0 at 25 DAG (A), 33 DAG (B), 41DAG (C) and 59 DAG (D).
(TIF)

Figure S3 **Rosette leaf number calculation.** The top views of Col-0, *skb1*, *35S::SKB1* *skb1* seedlings under a dissecting microscope at 6 DAG and 10 DAG. All mature rosette leaves as well as young leaves were counted. The arrowhead indicates a rosette leaf and c indicates a cotyledon.
(TIF)

Figure S4 **Phenotypes of *35S::SKB1 clv3-2* and *35S::SKB1 clv1-1*.** (A) Top views of inflorescences of L*er*, *35S::SKB1* L*er*, *clv3-2*, *35S::SKB1* *clv3-2*, *clv1-1*, *35S::SKB1* *clv1-1*.
(B) Phenotypes of flowers in *35S::SKB1* *clv3-2* and *35S::SKB1* *clv1-1* compared with L*er, clv3-2* and *clv1-1*.
(C) Phenotypes of whole plants of *35S::SKB1* *clv3-2* and *35S::SKB1* *clv1-1* compared with L*er, clv3-2* and *clv1-1*.
(TIF)

Figure S5 **ChIP analysis of Col-0 and *skb1* at the *WUS* locus.** (A) A diagram of the *WUS* gene structure, with bars representing the a-i regions examined by ChIP. White boxes indicate *WUS* open reading frame.
(B) The ChIP assay was performed with antibody against SKB1.
(TIF)

Figure S6 **Comparison of size of *crn* and *crn skb1* seedlings at 9DAG.** Scale bar = 1 cm.
(TIF)

Table S1 **primers used in the paper.**
(DOC)

## References

[1] Bowman JL, Eshed Y (2000) Formation and maintenance of the shoot apical meristem. Trends Plant Sci 5: 110-115. doi:10.1016/S1360-1385(00)01569-7. PubMed: 10707076. 10707076

[2] Lenhard M, Laux T (1999) Shoot meristem formation and maintenance. Curr Opin Plant Biol 2: 44-50. doi:10.1016/S1369-5266(99)80009-0. PubMed: 10047572. 10047572

[3] Fletcher JC (2002) Shoot and floral meristem maintenance in Arabidopsis. Annu Rev Plant Biol 53: 45-66. doi:10.1146/annurev.arplant.53.092701.143332. PubMed: 12221985. 12221985

[4] Mayer KF, Schoof H, Haecker A, Lenhard M, Jürgens G et al. (1998) Role of WUSCHEL in regulating stem cell fate in the Arabidopsis shoot meristem. Cell 95: 805-815. doi:10.1016/S0092-8674(00)81703-1. PubMed: 9865698. 9865698

[5] Laux T, Mayer KF, Berger J, Jürgens G (1996) The WUSCHEL gene is required for shoot and floral meristem integrity in Arabidopsis. Development 122: 87-96. PubMed: 8565856. 8565856 10.1242/dev.122.1.87

[6] Yadav RK, Perales M, Gruel J, Girke T, Jönsson H et al. (2011) WUSCHEL protein movement mediates stem cell homeostasis in the Arabidopsis shoot apex. Genes Dev 25: 2025-2030. doi:10.1101/gad.17258511. PubMed: 21979915. 21979915 PMC3197201

[7] Fletcher JC, Brand U, Running MP, Simon R, Meyerowitz EM (1999) Signaling of cell fate decisions by CLAVATA3 in Arabidopsis shoot meristems. Science 283: 1911-1914. doi:10.1126/science.283.5409.1911. PubMed: 10082464. 10082464

[8] Ohyama K, Shinohara H, Ogawa-Ohnishi M, Matsubayashi Y (2009) A glycopeptide regulating stem cell fate in Arabidopsis thaliana. Nat Chem Biol 5: 578-580. doi:10.1038/nchembio.182. PubMed: 19525968. 19525968

[9] Clark SE, Running MP, Meyerowitz EM (1995) CLAVATA3 is a specific regulator of shoot and floral meristem development affecting the same processes as CLAVATA1. Development 121: 2057-2067.

[10] Schoof H, Lenhard M, Haecker A, Mayer KF, Jürgens G et al. (2000) The stem cell population of Arabidopsis shoot meristems in maintained by a regulatory loop between the CLAVATA and WUSCHEL genes. Cell 100: 635-644. doi:10.1016/S0092-8674(00)80700-X. PubMed: 10761929. 10761929

[11] Brand U, Fletcher JC, Hobe M, Meyerowitz EM, Simon R (2000) Dependence of stem cell fate in Arabidopsis on a feedback loop regulated by CLV3 activity. Science 289: 617-619. doi:10.1126/science.289.5479.617. PubMed: 10915624. 10915624

[12] Clark SE, Williams RW, Meyerowitz EM (1997) The CLAVATA1 gene encodes a putative receptor kinase that controls shoot and floral meristem size in Arabidopsis. Cell 89: 575-585. doi:10.1016/S0092-8674(00)80239-1. PubMed: 9160749. 9160749

[13] Jeong S, Trotochaud AE, Clark SE (1999) The Arabidopsis CLAVATA2 gene encodes a receptor-like protein required for the stability of the CLAVATA1 receptor-like kinase. Plant Cell 11: 1925-1934. doi:10.2307/3871087. PubMed: 10521522. 10521522 PMC144110

[14] Trotochaud AE, Hao T, Wu G, Yang Z, Clark SE (1999) The CLAVATA1 receptor-like kinase requires CLAVATA3 for its assembly into a signaling complex that includes KAPP and a Rho-related protein. Plant Cell 11: 393-406. doi:10.2307/3870868. PubMed: 10072399. 10072399 PMC144183

[15] Ogawa M, Shinohara H, Sakagami Y, Matsubayashi Y (2008) Arabidopsis CLV3 peptide directly binds CLV1 ectodomain. Science 319: 294. doi:10.1126/science.1150083. PubMed: 18202283. 18202283

[16] Müller R, Bleckmann A, Simon R (2008) The receptor kinase CORYNE of Arabidopsis transmits the stem cell-limiting signal CLAVATA3 independently of CLAVATA1. Plant Cell 20: 934-946. doi:10.1105/tpc.107.057547. PubMed: 18381924. 18381924 PMC2390746

[17] Guo Y, Han L, Hymes M, Denver R, Clark SE (2010) CLAVATA2 forms a distinct CLE-binding receptor complex regulating Arabidopsis stem cell specification. Plant J 63: 889-900. doi:10.1111/j.1365-313X.2010.04295.x. PubMed: 20626648. 20626648 PMC2974754

[18] Bleckmann A, Weidtkamp-Peters S, Seidel CAM, Simon R (2010) Stem cell signaling in Arabidopsis requires CRN to localize CLV2 to the plasma membrane. Plant Physiol 152: 166-176. doi:10.1104/pp.109.149930. PubMed: 19933383. 19933383 PMC2799354

[19] Zhu Y, Wang Y, Li R, Song X, Wang Q et al. (2010) Analysis of interactions among the CLAVATA3 receptors reveals a direct interaction between CLAVATA2 and CORYNE in Arabidopsis. Plant J 61: 223-233. PubMed: 19843317. 19843317 10.1111/j.1365-313X.2009.04049.x

[20] Kinoshita A, Betsuyaku S, Osakabe Y, Mizuno S, Nagawa S et al. (2010) RPK2 is an essential receptor-like kinase that transmits the CLV3 signal in Arabidopsis. Development 137: 3911-3920. doi:10.1242/dev.048199. PubMed: 20978082. 20978082

[21] Betsuyaku S, Takahashi F, Kinoshita A, Miwa H, Shinozaki K et al. (2011) Mitogen-activated protein kinase regulated by the CLAVATA receptors contributes to shoot apical meristem homeostasis. Plant Cell Physiol 52: 14-29. doi:10.1093/pcp/pcq157. PubMed: 20965998. 20965998 PMC3023851

[22] Ahmad A, Cao X (2012) Plant PRMTs broaden the scope of arginine methylation. J Genet Genomics 39: 195-208. doi:10.1016/j.jgg.2012.04.001. PubMed: 22624881. 22624881

[23] Bedford MT, Richard S (2005) Arginine methylation an emerging regulator of protein function. Mol Cell 18: 263-272. doi:10.1016/j.molcel.2005.04.003. PubMed: 15866169. 15866169

[24] Bedford MT, Clarke SG (2009) Protein arginine methylation in mammals: who, what, and why. Mol Cell 33: 1-13. doi:10.1016/j.molcel.2008.12.013. PubMed: 19150423. 19150423 PMC3372459

[25] Pahlich S, Zakaryan RP, Gehring H (2006) Protein arginine methylation: Cellular functions and methods of analysis. Biochim Biophys Acta 1764: 1890-1903. doi:10.1016/j.bbapap.2006.08.008. PubMed: 17010682. 17010682

[26] Liu C, Lu F, Cui X, Cao X (2010) Histone methylation in higher plants. Annu Rev Plant Biol 61: 395-420. doi:10.1146/annurev.arplant.043008.091939. PubMed: 20192747. 20192747

[27] Pal S, Yun R, Datta A, Lacomis L, Erdjument-Bromage H et al. (2003) mSin3A/histone deacetylase 2- and PRMT5-containing Brg1 complex is involved in transcriptional repression of the Myc target gene cad. Mol Cell Biol 23: 7475-7487. doi:10.1128/MCB.23.21.7475-7487.2003. PubMed: 14559996. 14559996 PMC207647

[28] Kwak YT, Guo J, Prajapati S, Park KJ, Surabhi RM et al. (2003) Methylation of SPT5 regulates its interaction with RNA polymerase II and transcriptional elongation properties. Mol Cell 11: 1055-1066. doi:10.1016/S1097-2765(03)00101-1. PubMed: 12718890. 12718890

[29] Chari A, Golas MM, Klingenhäger M, Neuenkirchen N, Sander B et al. (2008) An assembly chaperone collaborates with the SMN complex to generate spliceosomal SnRNPs. Cell 135: 497-509. doi:10.1016/j.cell.2008.09.020. PubMed: 18984161. 18984161

[30] Zhou Z, Sun X, Zou Z, Sun L, Zhang T et al. (2010) PRMT5 regulates Golgi apparatus structure through methylation of the golgin GM130. Cell Res 20: 1023-1033. doi:10.1038/cr.2010.56. PubMed: 20421892. 20421892

[31] Guo S, Bao S (2010) srGAP2 arginine methylation regulates cell migration and cell spreading through promoting dimerization. J Biol Chem 285: 35133-35141. doi:10.1074/jbc.M110.153429. PubMed: 20810653. 20810653 PMC2966127

[32] Deng X, Gu L, Liu C, Lu T, Lu F et al. (2010) Arginine methylation mediated by the Arabidopsis homolog of PRMT5 is essential for proper pre-mRNA splicing. Proc Natl Acad Sci U S A 107: 19114-19119. doi:10.1073/pnas.1009669107. PubMed: 20956294. 20956294 PMC2973915

[33] Wang X, Zhang Y, Ma Q, Zhang Z, Xue Y et al. (2007) SKB1-mediated symmetric dimethylation of histone H4R3 controls flowering time in Arabidopsis. EMBO J 26: 1934-1941. doi:10.1038/sj.emboj.7601647. PubMed: 17363895. 17363895 PMC1847673

[34] Zhang Z, Zhang S, Zhang Y, Wang X, Li D et al. (2011) Arabidopsis floral initiator SKB1 confers high salt tolerance by regulating transcription and pre-mRNA splicing through altering histone H4R3 and small nuclear ribonucleoprotein LSM4 methylation. Plant Cell 23: 396-411. doi:10.1105/tpc.110.081356. PubMed: 21258002. 21258002 PMC3051234

[35] Pei Y, Niu L, Lu F, Liu C, Zhai J et al. (2007) Mutations in the Type II protein arginine methyltransferase AtPRMT5 result in pleiotropic developmental defects in Arabidopsis. Plant Physiol 144: 1913-1923. doi:10.1104/pp.107.099531. PubMed: 17573539. 17573539 PMC1949897

[36] Sanchez SE, Petrillo E, Beckwith EJ, Zhang X, Rugnone ML et al. (2010) A methyl transferase links the circadian clock to the regulation of alternative splicing. Nature 468: 112-118. doi:10.1038/nature09470. PubMed: 20962777. 20962777

[37] Carles CC, Fletcher JC (2003) Shoot apical meristem maintenance: the art of a dynamic balance. Trends Plant Sci 8: 394-401. doi:10.1016/S1360-1385(03)00164-X. PubMed: 12927973. 12927973

[38] Clark SE (2001) Cell signalling at the shoot meristem. Nat Rev Mol Cell Biol 2: 276-284. doi:10.1038/35067079. PubMed: 11283725. 11283725

[39] He Y, Amasino RM (2005) Role of chromatin modification in flowering-time control. Trends Plant Sci 10: 30-35. doi:10.1016/j.tplants.2004.11.003. PubMed: 15642521. 15642521

[40] Zuo J, Niu QW, Frugis G, Chua NH (2002) The WUSCHEL gene promotes vegetative-to-embryonic transition in Arabidopsis. Plant J 30: 349-359. doi:10.1046/j.1365-313X.2002.01289.x. PubMed: 12000682. 12000682

[41] Bäurle I, Laux T (2005) Regulation of WUSCHEL transcription in the stem cell niche of the Arabidopsis shoot meristem. Plant Cell 17: 2271-2280. doi:10.1105/tpc.105.032623. PubMed: 15980263. 15980263 PMC1182488

[42] Liu X, Kim YJ, Müller R, Yumul RE, Liu C et al. (2011) AGAMOUS terminates floral stem cell maintenance in Arabidopsis by directly repressing WUSCHEL through recruitment of Polycomb group proteins. Plant Cell 23: 3654-3670. doi:10.1105/tpc.111.091538. PubMed: 22028461. 22028461 PMC3229141

[43] Li W, Liu H, Cheng ZJ, Su YH, Han HN et al. (2011) DNA methylation and histone modifications regulate de novo shoot regeneration in Arabidopsis by modulating WUSCHEL expression and auxin signaling. PLoS Genet 7: e1002243. doi:10.1371/journal.pgen.1002243. PubMed: 21876682. 21876682 PMC3158056

[44] Barton MK, Poethig RS (1993) Formation of the shoot apical meristem in Arabidopsis thaliana: an analysis of development in the wild type and in the shoot meristemless mutant. Development 119: 823-831.

[45] Endrizzi K, Moussian B, Haecker A, Levin JZ, Laux T (1996) The SHOOT MERISTEMLESS gene is required for maintenance of undifferentiated cells in Arabidopsis shoot and floral meristems and acts at a different regulatory level than the meristem genes WUSCHEL and ZWILLE. Plant J 10: 967-979. doi:10.1046/j.1365-313X.1996.10060967.x. PubMed: 9011081. 9011081

[46] Byrne ME, Barley R, Curtis M, Arroyo JM, Dunham M et al. (2000) Asymmetric leaves1 mediates leaf patterning and stem cell function in Arabidopsis. Nature 408: 967-971. doi:10.1038/35050091. PubMed: 11140682. 11140682

[47] Tsiantis M, Hay A (2003) Comparative plant development: the time of the leaf? Nat Rev Genet 4: 169-180. doi:10.1038/nrg1002. PubMed: 12610522. 12610522

[48] Gilbreth M, Yang P, Wang D, Frost J, Polverino A, et al. (1996) The highly conserved skb1 gene encodes a protein that interacts with Shk1, a fission yeast Ste20/PAK homolog. Proc Nat lAcad Sci USA 93: 13802-13807. 10.1073/pnas.93.24.13802 PMC19432 8943016

[49] Gilbreth M, Yang P, Bartholomeusz G, Pimental RA, Kansra S et al. (1998) Negative regulation of mitosis in fission yeast by the shk1 interacting protein skb1 and its human homolog, Skb1Hs. Proc Natl Acad Sci U S A 95: 14781-14786. doi:10.1073/pnas.95.25.14781. PubMed: 9843966. 9843966 PMC24526

[50] Karkhanis V, Hu YJ, Baiocchi RA, Imbalzano AN, Sif S (2011) Versatility of PRMT5-induced methylation in growth control and development. Trends Biochem Sci 36: 633-641. doi:10.1016/j.tibs.2011.09.001. PubMed: 21975038. 21975038 PMC3225484

[51] Lenhard M, Bohnert A, Jürgens G, Laux T (2001) Termination of stem cell maintenance in Arabidopsis floral meristems by interactions between WUSCHEL and AGAMOUS. Cell 105: 805-814. doi:10.1016/S0092-8674(01)00390-7. PubMed: 11440722. 11440722

[52] Clark SE, Running MP, Meyerowitz EM (1993) CLAVATA1, a regulator of meristem and flower development in Arabidopsis. Development 119: 397-418. PubMed: 8287795. 8287795 10.1242/dev.119.2.397

[53] Miwa H, Betsuyaku S, Iwamoto K, Kinoshita A, Fukuda H et al. (2008) The receptor-like kinase SOL2 mediates CLE signaling in Arabidopsis. Plant Cell Physiol 49: 1752-1759. doi:10.1093/pcp/pcn148. PubMed: 18854335. 18854335 PMC2582179

[54] Scarpella E, Francis P, Berleth T (2004) Stage-specific markers define early steps of procambium development in Arabidopsis leaves and correlate termination of vein formation with mesophyll differentiation. Development 131: 3445-3455. doi:10.1242/dev.01182. PubMed: 15226260. 15226260

[55] Bowler C, Benvenuto G, Laflamme P, Molino D, Probst AV et al. (2004) Chromatin techniques for plant cells. Plant J 39: 776-789. doi:10.1111/j.1365-313X.2004.02169.x. PubMed: 15315638. 15315638

