# Supplementary figures and images for: Histone H4R3 Methylation Catalyzed by SKB1/PRMT5 Is Required for Maintaining Shoot Apical Meristem
Source: PLoS One. 2013 Dec 12;8(12):e83258. doi: 10.1371/journal.pone.0083258 (PMC3861506; doi:10.1371/journal.pone.0083258)

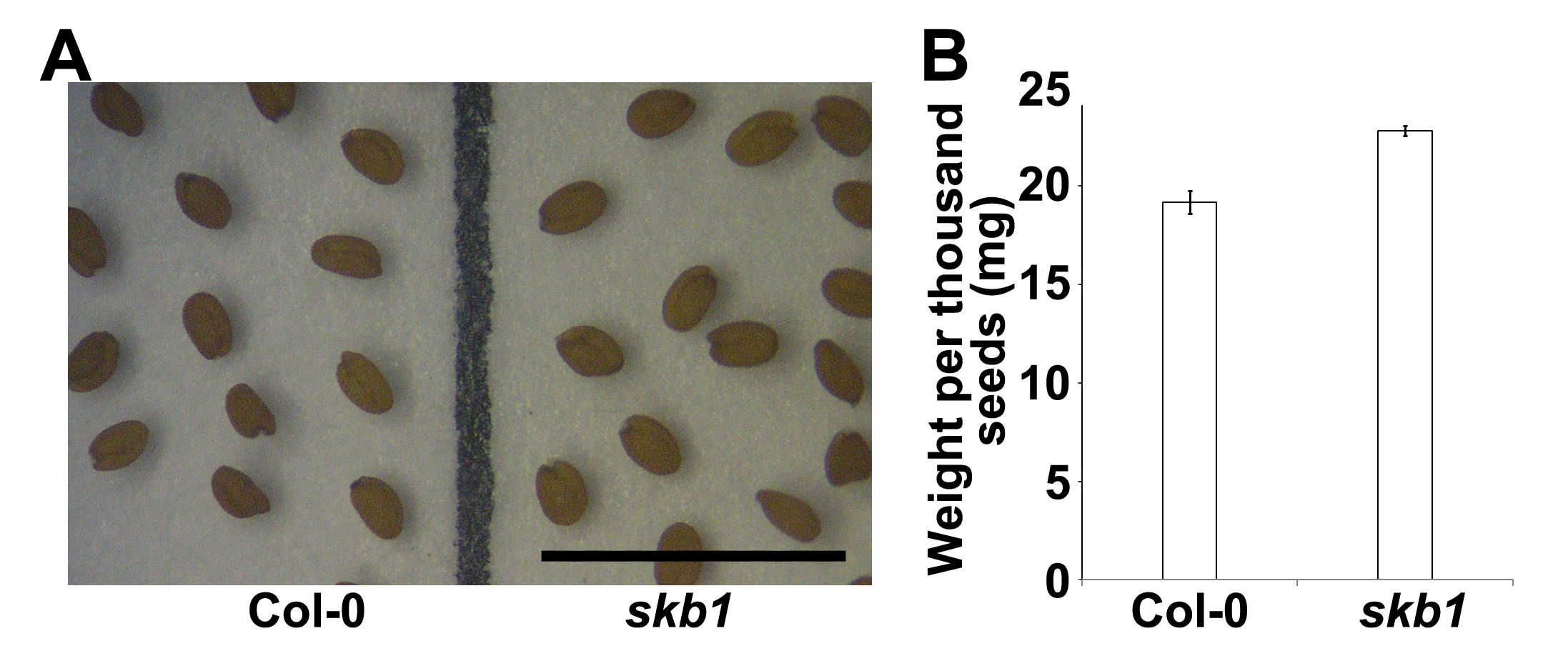

Supplement: Figure S1 — Comparison of skb1 and Col-0 seeds. (A) Morphology of skb1 and Col-0 seeds. (B) Comparison of weight per thousand seeds of skb1 and Col-0. (TIF) [file pone.0083258.s001.tif]

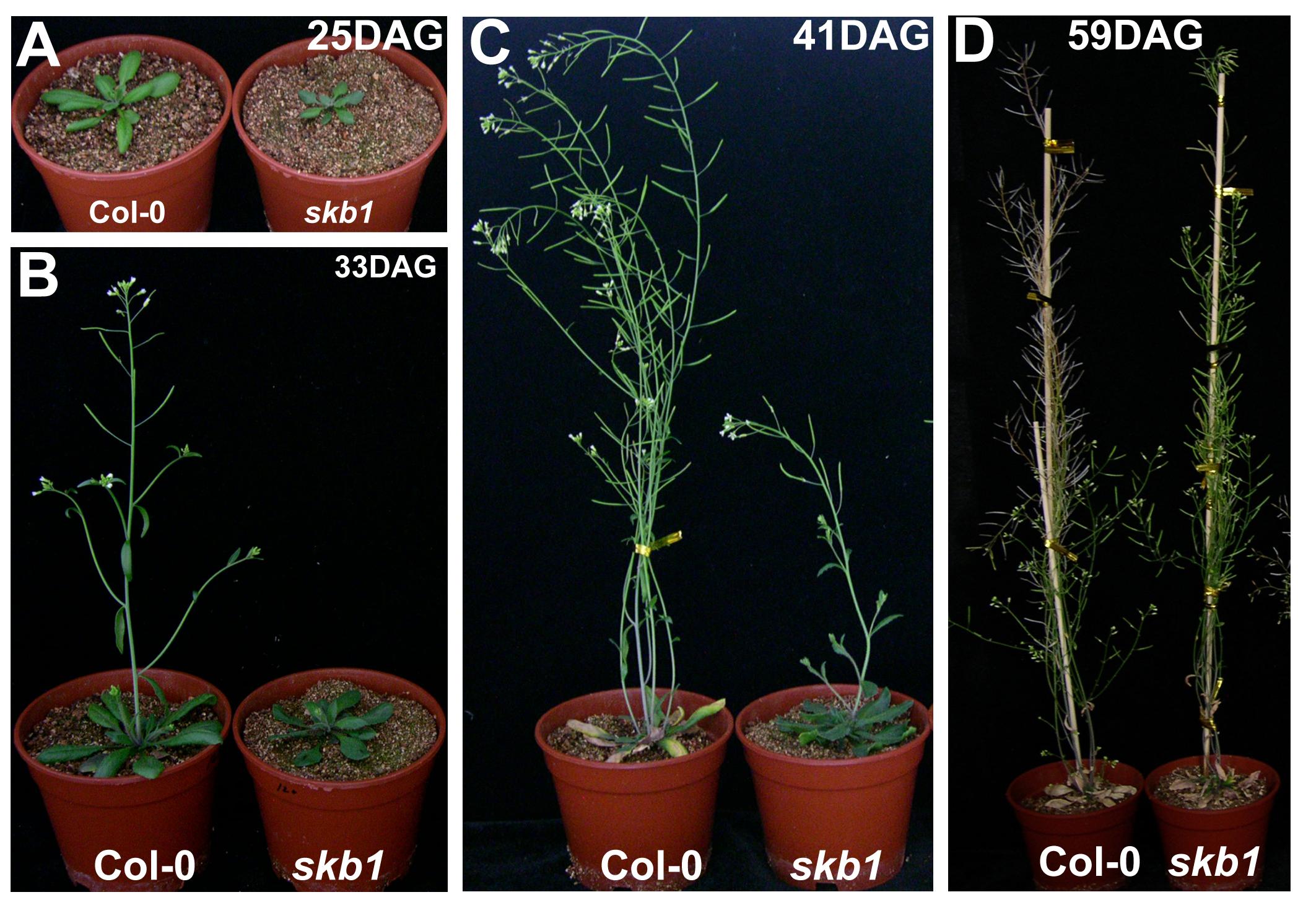

Supplement: Figure S2 — Phenotypes of skb1 in different growth stage. (A) to (D) Sizes of skb1 compared with Col-0 at 25 DAG (A), 33 DAG (B), 41DAG (C) and 59 DAG (D). (TIF) [file pone.0083258.s002.tif]

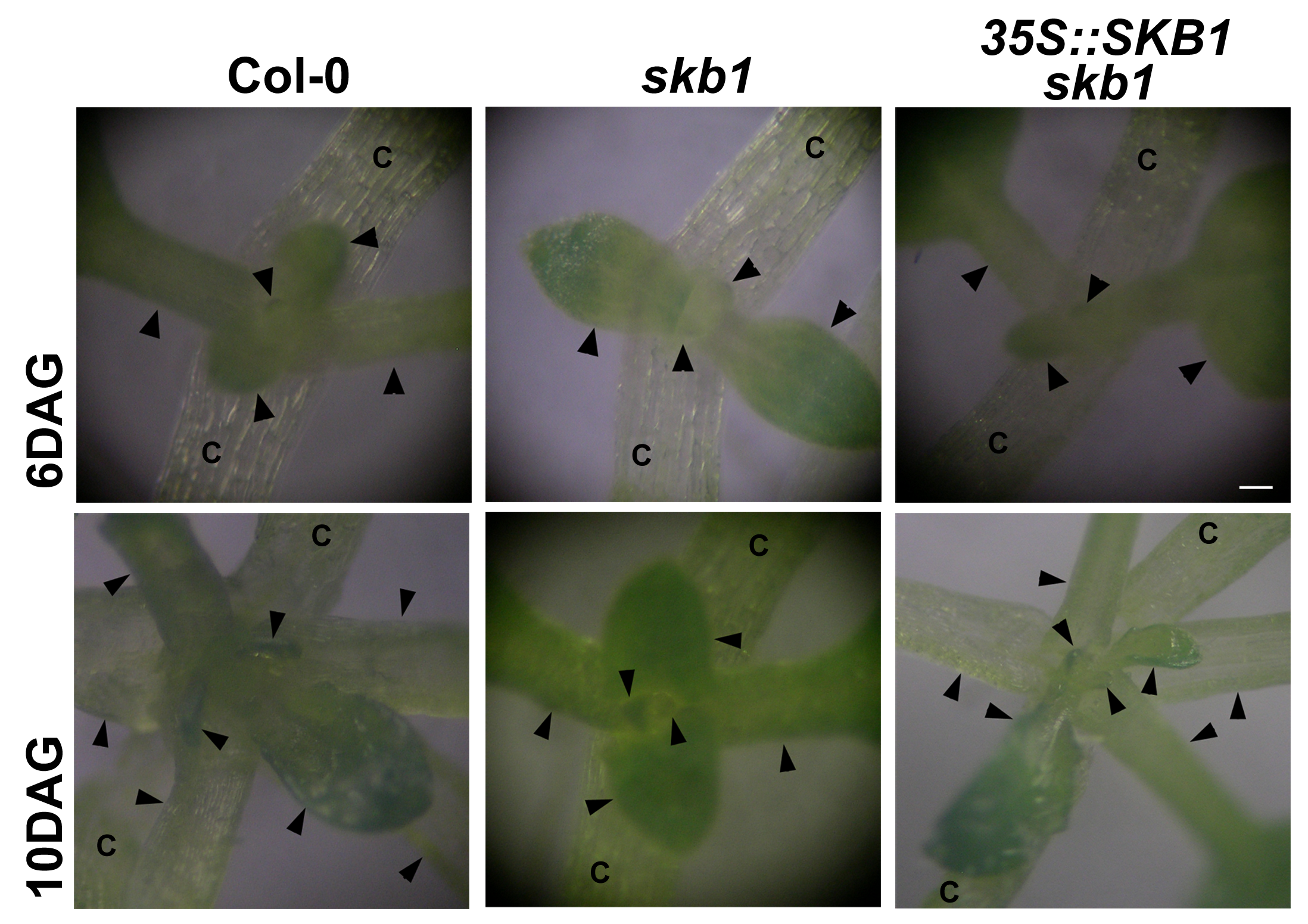

Supplement: Figure S3 — Rosette leaf number calculation. The top views of Col-0, skb1, 35S::SKB1 skb1 seedlings under a dissecting microscope at 6 DAG and 10 DAG. All mature rosette leaves as well as young leaves were counted. The arrowhead indicates a rosette leaf and c indicates a cotyledon. (TIF) [file pone.0083258.s003.tif]

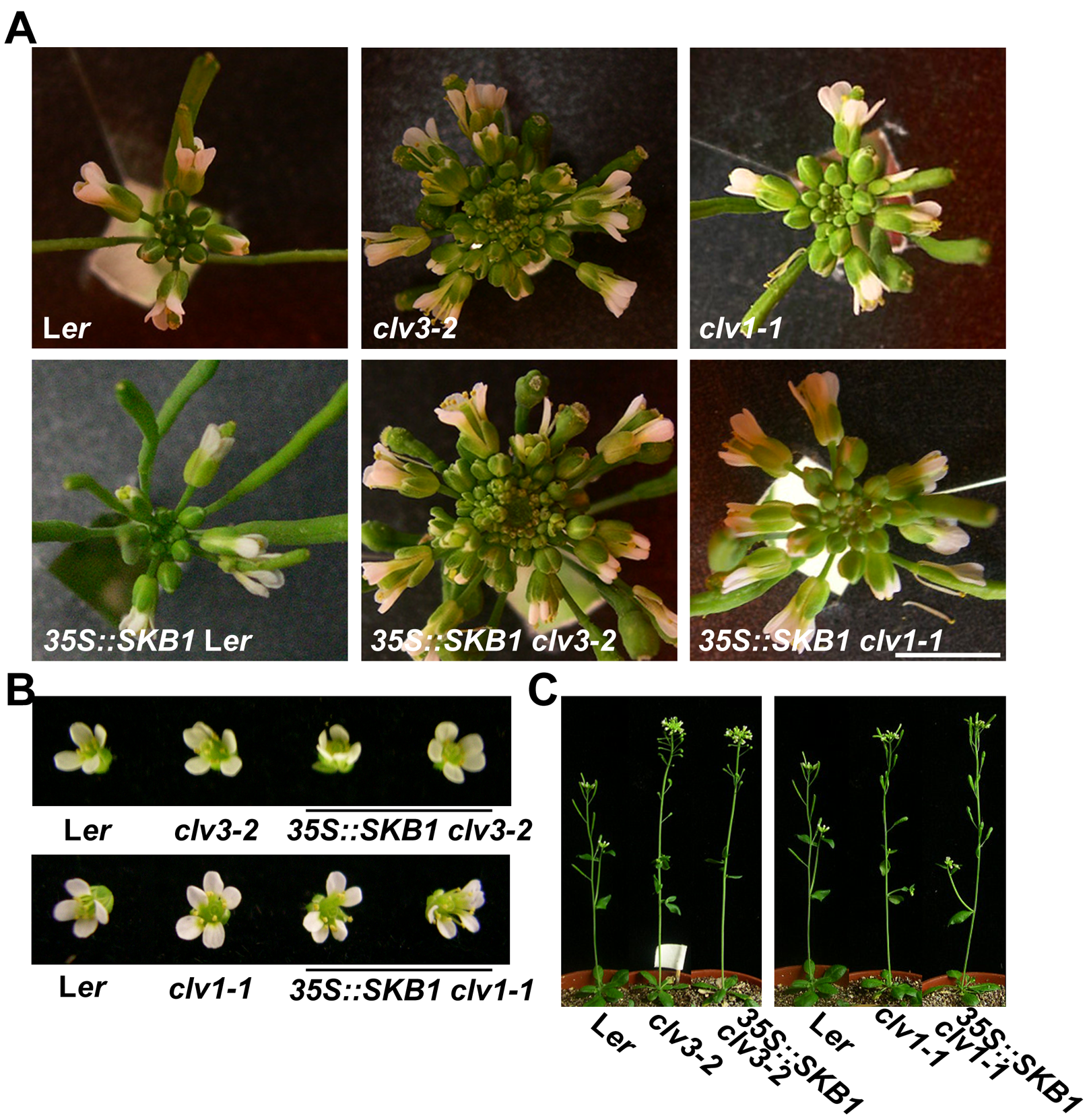

Supplement: Figure S4 — Phenotypes of 35S::SKB1 clv3-2 and 35S::SKB1 clv1-1. (A) Top views of inflorescences of Ler, 35S::SKB1 Ler, clv3-2, 35S::SKB1 clv3-2, clv1-1, 35S::SKB1 clv1-1. (B) Phenotypes of flowers in 35S::SKB1 clv3-2 and 35S::SKB1 clv1-1 compared with Ler, clv3-2 and clv1-1. (C) Phenotypes of whole plants of 35S::SKB1 clv3-2 and 35S::SKB1 clv1-1 compared with Ler, clv3-2 and clv1-1. (TIF) [file pone.0083258.s004.tif]

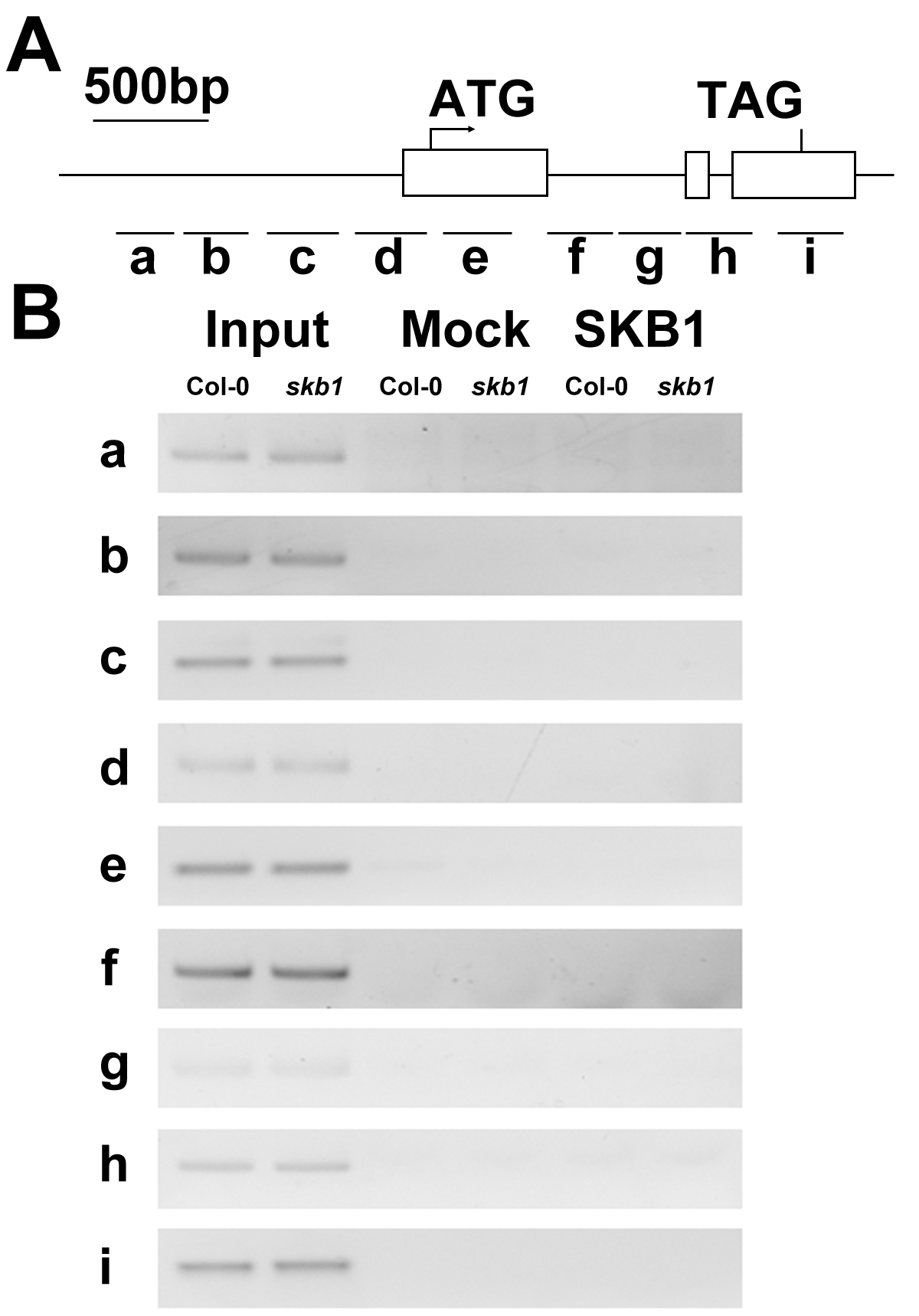

Supplement: Figure S5 — ChIP analysis of Col-0 and skb1 at the WUS locus. (A) A diagram of the WUS gene structure, with bars representing the a-i regions examined by ChIP. White boxes indicate WUS open reading frame. (B) The ChIP assay was performed with antibody against SKB1. (TIF) [file pone.0083258.s005.tif]

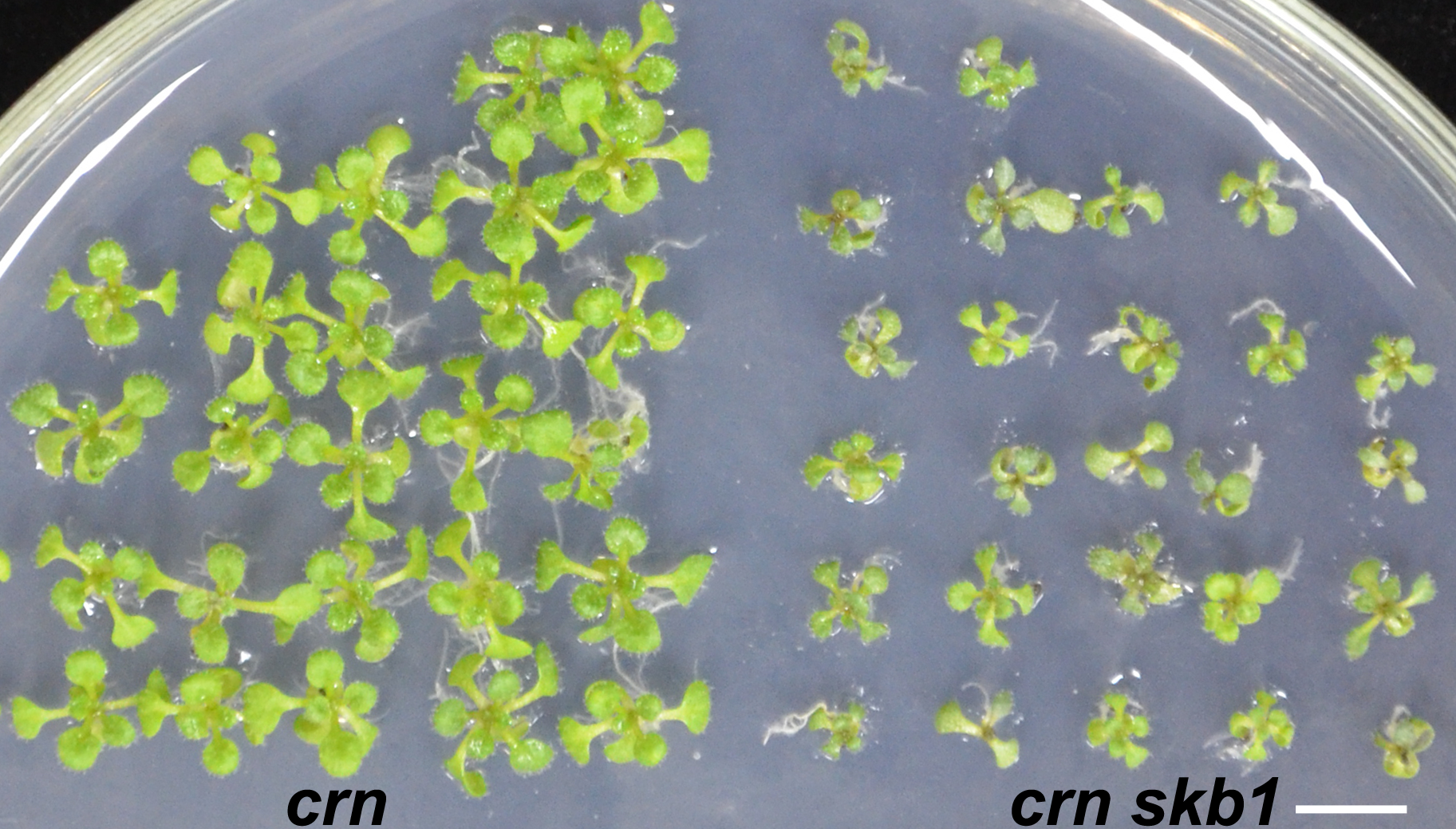

Supplement: Figure S6 — Comparison of size of crn and crn skb1 seedlings at 9DAG. Scale bar = 1 cm. (TIF) [file pone.0083258.s006.tif]
