# Supplementary material for: Histone H4R3 Methylation Catalyzed by SKB1/PRMT5 Is Required for Maintaining Shoot Apical Meristem
Source: PLoS One. 2013 Dec 12;8(12):e83258. doi: 10.1371/journal.pone.0083258 (PMC3861506; doi:10.1371/journal.pone.0083258)
Supplement: Table S1 — primers used in the paper. (DOC) [file pone.0083258.s007.doc]

| **Semi-quantitative RT–PCR primers** | |
| --- | --- |
| *STM* | LP：CATAACGAAATCGTTGCAGGA |
|  | RP：ATCGACTTCTTCCTCGGATGA |
| *KNAT1* | LP：TGTCAGAGTCCCATTCAC |
|  | RP：GCAACGAGAGGTTGTTATT |
| *KNAT2* | LP：TGACGAGGAACTGAGAGAAG |
|  | RP：GGTTCCATTCATTCGCGATG |
| *KNAT6* | LP：GGTGTAATATCATCTGACG |
|  | RP：TGTCGTCCATGCTTCACTC |
| *WUS* | LP：TAACAAGCCATATCCCAGC |
|  | RP：GCTTTAATCCCGAGCGAC |
| *CLV3* | LP：TGTACTCATTAAGGCCTCTC |
|  | RP：CATGAAACTACAAGCTTACC |
| *CLV1* | LP：GCTTGCTAAGTTCTTAGTTGATGG |
|  | RP：GCATCCGATGGCTGAGTTAT |
| *CLV2* | LP：TCGATACCCGCCACATTTGTAAGTC |
|  | RP：GAGAGAGGTCAAGAAGAACCAAACCC |
| *AG* | LP：AATCAGCCAAATTGCGTCAAC |
|  | RP：GCTTTATATTGCTTGCTCAACC |
| *AP3* | LP：GAGACAAATAGAAATCTCCG |
|  | RP：TAGACAATGATGGCACCAGC |
| *AS1* | LP：GAAGTTGCTCTTGAGTTTGGG |
|  | RP：GCTCAACTCTCTTGTTACTC |
| *AS2* | LP：CCTCTGAGCAACAGAAGCC |
|  | RP：CCGAGGCTTTGGTACTTAG |
| *POL* | LP：GTACCACCGACCGTAGTGCT |
|  | RP：GTGACGATCATGGAGCCTTT |
| *TUBULIN* | LP：TTTGGAGCCTGGGACTATGGAC |
|  | RP：ACGGGGGAATGGGATGAGAT |
| **Qualitative real time PCR primers** | |
| *WUS* | LP：GGTCACCAACAGCCGATCA |
|  | RP：AAGACGTTCTTGCCCTCAATCT |
| *CLV3* | LP：CACTCAAGCTCATGCTCACGTT |
|  | RP：CCCATTCACTTTCCATTTTCATC |
| *CRN* | LP：GAGTCCTCTCAAAGTAACAGATACACAGA |
|  | RP：TCCGGTTAAAAGAACACCCAAT |
| *ACTIN2* | LP：GGTAACATTGTGCTCAGTGGTGG |
|  | RP：AACGACCTTAATCTTCATGCTGC |
| **ChIP primers** | |
| *WUS-a* | LP：TATCAGCCGTATCTTCAGGGCCA |
|  | RP：TGCTCGAATTTATAGGAGCATGGATG |
| *WUS-b* | LP：TCAACATGTTCATAAGTACACCTGTCTTCAC |
|  | RP：ATTGTATCGTAGGAATGAAAGAAAACGATT |
| *WUS-c* | LP：GACAGATACATAACAATTAAAGCTGGTGAAGT |
|  | RP：GGAAGGAAATTCTCTTGTCAAATATATTATTTGA |
| *WUS-d* | LP：TATCCTTTTCTTTGGCAAAATTTATTTCTTAT |
|  | RP：ACTTTTGTTCACAAAGTTAACGGTAAATAAGAT |
| *WUS-e* | LP：CAAGAAAGCGGCAACAACAACAAC |
|  | RP：AGTTGGGTGATGAAGATGGTGTGGT |
| *WUS-f* | LP：GTGACTAATCTCTCTTATACATGCTTTTTTATGAA |
|  | RP：CATGTAATAATAGATGGTGTGTAACATGTTCCTA |
| *WUS-g* | LP：CCAAGAAGATGTTATGTACATGTAATGACGTA |
|  | RP：TCATCAGTGATCATATATGTTGTGATTCCAG |
| *WUS-h* | LP：GAATTTAAATCATGCAAGCTCAGGTACTG |
|  | RP：CAAACAGAGGCTTTGCTCTATCGAAGA |
| *WUS-i* | LP：GTGCCATCTGGAAGTATGGCCAA |
|  | RP：CTTATTCATCATAGAGATAAAACGGTTGTCAA |
| *CRN-a* | LP：TGTATTGATGTTTTTGTGATGGATG |
|  | RP：TACAAGTTACAACGTCTGGACATGA |
| *CRN-b* | LP：TCTCAGACAAACAAAGGAACCACT |
|  | RP：AGATGCGGTTAGTGAGATATGGAC |
| *CRN-c* | LP：TTTATTTTGGAGGAAAAGAGTGAGA |
|  | RP：CCAATGAGAAACCACCAAGACA |
| *CRN-d* | LP：CCAGAAAGTAGTAGTAGCAAGTCGG |
|  | RP：TCATAGACCAGAGAGAACTCATCAGA |
| *CRN-e* | LP：AAAACCTCTGTAAAACCAATCCA |
|  | RP：TTTTTGTACCATTTGCGTGATC |
